# Designing the Antioxidant Properties of Low-Processed Food

**DOI:** 10.3390/antiox9100975

**Published:** 2020-10-12

**Authors:** Michał Świeca

**Affiliations:** Department of Biochemistry and Food Chemistry, University of Life Sciences, Skromna Str. 8, 20-704 Lublin, Poland; michal.swieca@up.lublin.pl; Tel.: +48-81-462-33-93

Food is the most valuable source of components exhibiting antioxidant properties. These ingredients belong to different groups, e.g., carotenoids, vitamins, phenolic compounds, peptides, and essential oils, but their common nature includes different modes of antioxidant action [1,2]. Antioxidants play an important role in homeostatic systems. They are responsible inter alia for maintaining redox status, can act as signaling compounds, or can be healing agents [3]. There are also many in vivo studies of the key roles of antioxidant activity in the treatment of many diseases, such as cardiovascular disorders, diabetes, cancers, or neurodegenerative disorders [2,3] Furthermore, antibacterial, antiviral, anti-inflammatory, and anticancer activities are usually related to antioxidant properties [4,5].

In modern communities, non- and low-processed foods represent an important branch of the market, including unprocessed, ready-to-eat snacks of fruit and vegetable, sprouted grains, and nuts, as well as minimally processed meals, e.g., mixed salads, juices, and smoothies. The composition and prohealth properties of food can be effectively shaped in each step of production; however, in the case of low-processed products, these modifications are limited to pre- and postharvest treatments of components or final products (e.g., biofortification, elicitation) and establishment of the conditions of storage.

The antioxidant potential of sprouted foods is effectively improved by elicitation with biotic (e.g., fungal cell wall components, plant hormones) and abiotic factors (e.g., metal ions, heating). Elicitors induce stress and disturb redox homeostasis, which usually results in de novo synthesis of antioxidants, e.g., phenolics and vitamins [6]. Pérez-Balibrea et al. [7] showed that chitosan, methyl jasmonate, salicylic acid, and yeast extract applications increased the content of phenolics, glucosinolates, and ascorbic acid in broccoli sprouts. Elicitation effectively improved antioxidant content in legumes [8,9,10], cereals [11,12], and cruciferous sprouts [13,14]. The final effect of elicitation can be additionally supported by precursor feeding, i.e., supplementation of growing plants with metabolite precursors of natural antioxidants. Phenylalanine, tyrosine, or shikimic acid feeding (precursors of phenolics synthesis in the phenylopropanoid pathway) was successfully applied to produce lentil [15], buckwheat [16], and broccoli [7] sprouts with enhanced antioxidant potential. Postharvest processing is focused on physicochemical treatments and/or the addition of exogenous antioxidants (or antioxidant-rich extracts) to directly improve antioxidant potential, reduce endogenous microbiota growth, and/or influence the activity of enzymatic systems responsible for antioxidant metabolism [17,18]. Minimal processing, e.g., cutting, makes disturbed tissues susceptible to microbiological contaminations and induces high endogenous enzyme activity (especially oxidases consuming phenolics). For this reason, low-processed foods are usually stored at low temperatures before consumption. All of these factors negatively influence the consumer quality of food while also reducing antioxidant proprieties. On the other hand, mechanical stress and chilling cause slight oxidative stress, acting as elicitor mechanisms. Additionally, these effects can be enhanced by further modifying storage conditions, e.g., illumination or chemical pretreatment. In the current literature, there are some studies covering the issues mentioned above. Consumer quality and low-molecular weight antioxidants can be effectively protected during storage through a modified atmosphere, as shown by Alasalvar and coworkers [19] in the case of ready-to-eat shredded orange and purple carrots, or by Fan and colleagues [20] in the case of fresh-cut iceberg lettuce. Matsufuji and coworkers successfully tailored microbiological quality and antioxidant potential of fresh-cut vegetables using nonthermal processes, including high-pressure carbon dioxide, gaseous chlorine dioxide, and pulsed xenon treatments [21]. Postharvest treatments with typical chemical solutions or biopreservatives, e.g., ascorbic acid [22], citric acid [23], or bacteriocins [24], were previously successfully used to improve antioxidant content during storage of minimally processed fruit and vegetables. Recently, some alternative approaches were been applied, e.g., the use of natural phenolic-rich solutions to improve the phenolic content and antioxidant capacity in stored mung bean sprouts and shredded iceberg lettuce [25,26]. Additionally, functional coatings [27] or enrichment with probiotics [9,28] turned out to be effective “tools”.

This Special Issue brings together valuable studies on tailoring antioxidant activity in low-processed food products. Collectively, this Special Issue contains seven empirical investigations, spanning the direct and indirect tools for tailoring the antioxidant capacities of low-processed foods. 

Dzugan and colleagues [29] developed a new dye from black elderberry fruits and flowers and studied its usefulness in improving the antioxidant potential of food. They showed that black elder fruits are rich in anthocyanins, especially cyanidin-3-*O*-sambubioside (7.56 mg/g dw), while flowers contain mainly chlorogenic acid (2.82 mg/g dw) and rutin (4.04 mg/g dw). Jellies enriched with functional components were characterized by favorable organoleptic properties and significantly higher antioxidant properties (ferric reducing/antioxidant power assay and DPPH radical scavenging tests). Spray-dried flavonoid-inulin microparticles designed as antioxidants for lipid systems were constructed by Morelo and coworkers [30]. They studied semicrystalline and amorphous microparticles of epicatechin and quercetin, with inulin formulated by spray-drying at two infeed temperatures (15 °C and 90 °C). The analyses showed that the quercetin–inulin microparticles possessed strong lipid-protecting proprieties, confirmed by the long-term experiment (35 days). However, these studies did not describe directly the antioxidant effects in low-processed foods, rather focusing on the development of functional products that can be easily applied to improve consumer quality. 

The antioxidant potential of sprouted food can be effectively tailored by germination conditions. Tomé-Sánchez and colleagues [31] examined the influence of germination temperature (12–21 °C) on soluble phenolic variation in wheat sprouts. Wheat germinated at 16 °C for seven days and 21 °C for four days accumulated the highest amounts of free phenolics (150 and 157 mg/100 g dw, respectively). In these samples, the phenolic fraction was composed of 57–58% phenolic acids, 38–39% flavonoids, and 3–5% lignans. The total antioxidant activity of dark-germinated sprouts was assayed using tests based on different mechanisms (ferric reducing/antioxidant power assay (FRAP), DPPH and ABTS radical scavenging tests, and oxygen radical absorbance capacity (ORAC)). The highest values in the ORAC (2.06–2.4 mmol Trolox equivalent/100 g dw), ABTS (2.9–3.0 mmol Trolox equivalent/100 g dw), FRAP (0.12–0.16 mmol Fe^2+^/g dw), and DPPH (0.32–3.72 mmol Trolox equivalent/100 g dw) assays were determined for six- and seven-day-old sprouts obtained at 16–21 °C. The highest anti-inflammatory properties (with the ability to inhibit TNF-alfa and IL-6 formation in RAW264.7 cells after LPS stimulation) were determined for four-day-old sprouts obtained at 16 °C (76.8% and 74.8% inhibition of TNF-alfa and IL-6, respectively). Finally, based on statistical analysis, it was found that the greatest improvement in the nutraceutical value of the wheat sprouts was achieved at 21 °C after seven days of sprouting. Effects of darkness and light spectra on nutrients and pigments in five-day-old radish, soybean, mung bean, and pumpkin sprouts were studied by Mastropasqua and coworkers [32]. They found that white, red, and blue light increased the contents of vitamin C, carotenoids, chlorophylls, and anthocyanins in all types of sprouts (compared to the control obtained in darkness). Surprisingly, there was no positive effect of the different illumination types on the total phenolics content, except soybean sprouts treated with red light; however, there was a significant ca. two-fold increase in ascorbic acid content in the case of pumpkin and mung bean sprouts treated with white light. On the other hand, these conditions significantly enhanced the utilization of stored materials, which was reflected in a subsequent loss of dry matter. The authors emphasized that the use of a specific spectral wavelength during sprouting could be an effective tool to improve the prohealth properties of final products. 

It is well-known that the antioxidant properties of food are strongly related to the activity of the endogenous enzymatic system. Two articles covered the effect of natural extracts on the enzymatic browning level resulting mainly from the oxidation of natural phenolic compounds by polyphenoloxidase and peroxidase in fresh-cut products. Sikora and colleagues [33] examined the effects of basil leaf (BLE) and wheat bran (WBE) extracts (potent antibrowning agents) on the phenolic content, antioxidant potential, microbiological quality, and consumer quality of shredded lettuce during storage. The treatment of lettuce with basil leaf extracts increased the total phenolic content and antioxidant properties without any negative influence on consumer quality. After eight days of storage (4 °C), the best results of the reducing potential and ability to quench radicals were found for the lettuce soaked in 1% (33 mg Trolox equivalent/g dm and 2.8 mg Trolox equivalent/g dm, respectively). The application of the functional solution to fresh-cut lettuce also improved the microbiological quality, with the coliform count reduced by 84% and 88% in samples treated with 0.5% BLE and 10% WBE, respectively. Natural extracts were tested as potential mitigators of fruit browning by Dias and colleagues [34]. Strawberry leaves and branches and an apple byproduct were selected for screening studies based on their high antioxidant properties. The leaves and branches of strawberry trees contained higher amounts of total phenolic content (208 mg/g and 104 mg/g, respectively) compared to the apple byproduct (6.7 mg/g). The authors found that the studied extracts were able to inhibit the activity of both polyphenoloxidase and peroxidase, with higher effectiveness against peroxidases. The antibrowning properties of the functional solution tested in cut pear discs exposed to air for 6 h showed that the extracts obtained from the strawberry branches and apple byproduct contributed to maintenance of the natural color of sliced fruit to the greatest extent. 

Consumer demand for minimally or nonprocessed food with preserved quality requires new strategies focused on extending the shelf-life of such products. In the study by Sachadyn-Król and coworkers [35], antioxidant properties of stored hot red pepper fruits were maintained and even improved by ozonation. The pepper fruits were ozonated for 1 and 3 h after harvesting and stored for 30 days in refrigeration conditions. The application of ozone for 3 h caused a significant increase in the content of total phenolic compounds. The best results were recorded on the 20th day after harvest in the samples treated for 3 h. In turn, after 30 days of storage, the ozonation treatment seemed to have a negative influence on phenolic content in the fruit pericarp; however, this effect was not reflected in antiradical properties. The study showed that ozone application can be a sufficient strategy to improve consumer quality of fruit and vegetables. 

Collectively, the antioxidant properties of low-processed foods can be effectively tailored. Many strategies can be employed to receive desirable results, with some examples presented in this Special Issue. Certainly, the “tools” should be customized for specific food products that usually differ from each other significantly in terms of form, type, content of bioactive components, shelf-life, and expected nutritional and physiological effects. Further studies are necessitated to elucidate the mechanisms of selected features and identify the key factors influencing the actions of antioxidants in food. 
